# Addressing sarcopenia management in primary care in Türkiye: gaps in the field and training needs of family physicians

**DOI:** 10.3389/fpubh.2025.1660782

**Published:** 2025-09-05

**Authors:** Ahmed Almatar, Şenay Koçakoğlu

**Affiliations:** Department of Family Medicine, Faculty of Medicine, Harran University, Şanlıurfa, Türkiye

**Keywords:** family physicians, sarcopenia, knowledge, management, older adults, muscle mass, muscle strength, physical performance

## Abstract

**Background and objectives:**

Sarcopenia is a current health threat that accompanies nutritional problems, cancers, and chronic diseases all around the world. Individuals in the risk group, especially the older adults, can be protected against sarcopenia with early diagnosis and effective interventions. The aim of this study is to conduct the first national survey to determine the awareness, knowledge, and adequacy levels of Turkish family physicians regarding sarcopenia management.

**Materials and methods:**

This cross-sectional study was performed on family physicians serving in family health centers across Türkiye. The mobile phone numbers of family physicians were accessed through the Federation of Family Physicians Associations. Data were collected through a 33-item online purpose-designed survey from April 1 to June 2024. The Kolmogorov–Smirnov test was used to evaluate the conformity of the variables to the normal distribution. Chi-square test was applied for comparisons between groups.

**Results:**

A total of 405 family physicians representing all seven regions in Türkiye participated in the study. The mean score of the participants’ competence level in sarcopenia management was found to be 3.37 ± 1.98 out of 10 points. Receiving training on palliative care and sarcopenia before and/or after graduation and having people diagnosed with sarcopenia in the immediate environment provided a positive and significant difference in terms of sarcopenia management competence scores (*p* < 0.001, *p* = 0.012, *p* = 0.001, respectively).

**Conclusion:**

The findings of the study reveal deficiencies in awareness, knowledge, and management of sarcopenia among family physicians. Expanding the subject of sarcopenia in the medical school curriculum, including sarcopenia management in guidelines for primary health care providers and providing equipment support, ensuring that family physicians are more proactive and competent in the management of sarcopenia may be a rational approach within the scope of protecting and improving individual and public health.

## 1 Introduction

Sarcopenia, as defined by the European Working Group on Sarcopenia Older People [EWGSOP (2018)], is a syndrome characterized by progressive and generalized loss of skeletal muscle mass and strength, and its diagnosis requires documentation of low muscle strength and low muscle mass. Poor physical performance is used as a criterion for classifying the severity of sarcopenia (1). Since sarcopenia includes both loss of muscle mass and muscle strength, it is considered in the category of muscle diseases with an ICD-10-CM Diagnosis Code (2).

Sarcopenia, which is seen in approximately 10–16% of the older population in the world, progresses without obvious symptoms at an early stage, like many chronic diseases; 15% of individuals over the age of 65 and 50% of those over the age of 80 suffer from sarcopenia (3). Sarcopenia, a major public health concern responsible for significant economic burden, is projected to become increasingly prevalent over the next 30 years. The older population represent the most commonly affected risk group (4, 5). Sarcopenia is a complex and progressive syndrome in which neurological, endocrine, and immune mechanisms play a role as well as aging. Ideal support of nutritional needs and muscle restructuring with resistance exercises are the most effective interventions in the treatment of sarcopenia, and it has been reported that studies on the use of drugs such as ACE inhibitors are ongoing (6, 7).

If the common decline in muscle mass with aging is overlooked and preventive measures are not implemented, it will inevitably lead to reduced physical performance, worsening balance problems, and an increased risk of falls (8–11). Nevertheless, by raising awareness and providing sufficient social and medical support, it is possible to promote healthy aging through the prevention of sarcopenia-related complications (12). Although it was previously defined only as loss of muscle mass due to aging, it has now been shown to accompany conditions like cancers and chronic diseases (e.g., cardiovascular and cerebrovascular). Insufficient protein-calorie intake, low physical activity levels can contribute to sarcopenia so it is important to closely monitor muscle strength and muscle mass in risk groups (13–15). As sarcopenia severity increases, individuals at risk experience reduced physical performance, limited mobility, balance issues, head and bodily injuries, bleeding, and fractures that can be fatal due to falls. It is reported that conditions such as obesity, malnutrition, and vitamin D deficiency are associated with the development of sarcopenia, a decrease in muscle mass causes an increase in fat mass, and insufficient protein and energy intake prepares the ground for malnutrition (16–18).

In aging world there appears to a need for primary health care professionals to take an active role in the management of sarcopenia. There are primary care studies on this subject around the world, and according to the current literature, the interest of researchers in this subject is increasing day by day (19, 20). Raising awareness, especially among health professionals, that sarcopenia can be prevented and treated through nutritional support, resistance training, and structured exercise will greatly improve quality of life and help reduce the overall healthcare burden (1, 7, 11, 21).

Providing health care services at the primary care level offers family physicians a valuable opportunity to prevent diseases and make early diagnosis. While structured training programs and clinical guidelines enhance family physicians’ ability to manage various conditions, sarcopenia remains underrecognized and undertreated.

Research suggests that many family physicians, despite being the initial healthcare contact for patients, have limited awareness and understanding of sarcopenia, contributing to its frequent underdiagnosis and insufficient management (19, 22). In this context, there is a need to increase the awareness and knowledge of family physicians about sarcopenia. In addition, providing the necessary equipment for the diagnosis of sarcopenia in primary care institutions can be a practical and cost-effective public health strategy to protect and promote health in aging societies. Due to the critical importance of early diagnosis and treatment of sarcopenia in terms of protecting and improving individual and community health, the availability of applicable and effective screening tools in primary care is crucial. However, many prominent studies in the literature highlight significant limitations that complicate this process in primary care. At this point, tools suitable for screening in primary health care settings are not sufficient (23). While the SARC-F questionnaire is frequently used due to its simplicity and low cost, and although basic questions (e.g., strength, assistance with walking, rising from a chair, climbing stairs, and falls) can be self-reported, it has been shown to lack sufficient sensitivity to detect sarcopenia in primary care settings (24). However, due to the high specificity of SARC-F, this tool was concluded to be preferred to use to exclude sarcopenia rather than to diagnose it (25).

One of the most important reasons why family physicians are not effective in the diagnosis of sarcopenia in primary care is the impracticality of the recommended diagnostic tools (26). Although dual-energy x-ray absorptiometry (DXA) to measure bone mineral density and assess body composition and bioelectrical impedance analysis (BIA) to assess body fat and lean mass are effective tools, their high cost, accessibility difficulties, and requirement of trained personnel make them impractical for use in primary care (27). Considering all these options, it may be more appropriate to use more practical tools such as hand grip test in primary care in sarcopenia assignment (28). The recently developed Sarcopenia Scoring Assessment Models (SarSA-Mod) are recommended as a viable option for diagnosis in primary care (29).

Since sarcopenia is often observed concurrently in many patients requiring palliative care, incorporating more comprehensive coverage of palliative care alongside sarcopenia in both undergraduate and postgraduate curricula may significantly enhance awareness and knowledge levels (30, 31).

This study aims to conduct the first survey to assess the awareness, knowledge, and competency levels of Turkish family physicians regarding sarcopenia management. The broader goal is to inform health authorities about the need for efficient strategies to raise sarcopenia awareness in primary care and improve management outcomes.

## 2 Materials and methods

### 2.1 Study design and sample selection

The study was approved by the Harran University Clinical Research Ethics Committee (18.03.2024–02/97). All the study procedures complied with the Helsinki Declaration.

This study was planned as cross-sectional design with the aim of evaluating knowledge, attitudes, and obstacles to the ideal management competence of the family physicians actively working in Family health Centers in Türkiye. Data were collected from April 1 to June 2024 through a 33-item online purpose-designed survey which is prepared by study team.

In Türkiye, there are approximately 8,163 family health centers (FHCs) operating under the primary healthcare system. These centers are widely distributed across both urban and rural regions, ensuring nationwide accessibility to primary care services. It is aimed to include family physicians working in FHCs located in various geographical areas, covering both urban and rural settings. By targeting this diversity, it is aimed to reflect the general distribution of family physicians and enhance the representativeness of the findings.

The sample size for the study was determined using the OpenEpi software. Based on a 5% type I error rate and a 95% confidence interval, the required sample size was calculated to be 380 participants. A total of 405 participants completed the survey by the end of the predetermined data collection period.

The inclusion criteria were based on a voluntary basis, and family physicians who agreed to participate in the study by convenience sampling method were included in the study (32). Access to family physicians was provided by contacting the Türkiye Family Physician’s Federation with the support and cooperation of the Şanlıurfa Family Physicians Association. The survey link was shared and forwarded to the communication groups of the family medicine associations and to the mobile phones of the individuals who could be accessed to ensure participation. At the beginning of the study participants were presented with an informational statement outlining the purpose of the study and were required to click “Yes” to provide their consent and proceed. Questions on the definition and diagnosis of sarcopenia were prepared based on the revised report of EWGSOP (2018), which is considered the gold standard in this regard (1).

### 2.2 Data collection tool and variables

The study data were collected through a purpose designed 33-item online survey created through the Google Forms Application. The data collection form consisted of nine items related to sociodemographic characteristics and 24 items to question the participants’ knowledge, attitudes, and barriers about ideal sarcopenia management, which was created by using the relevant guidelines and literature. Since there is no scale developed or validated in Türkiye on this subject, the data collection form was created by the researchers and no scores could be assigned to the answers of the participants. The survey was designed for descriptive purposes, comprising sociodemographic items and factual questions about sarcopenia.

The independent variables included demographic and professional factors such as age, gender, educational level, years of professional experience, specialist status, prior training in palliative care and sarcopenia, and whether participants had close contacts diagnosed with sarcopenia. The dependent variables consisted of three survey items designed to investigate participants’ knowledge and attitudes regarding sarcopenia management. These items were as follows: “Which of the following is the most reliable measure of muscle function in the diagnosis of sarcopenia?”; “How to identify sarcopenia?”; “How would you rate your level of competence in managing sarcopenia?”

Data collection form was not intended as a scale, and no composite or latent variables were derived from correct responses. Responses to all items were summarized using counts (*n*) and percentages (%). In only one item, participants were asked to assign a self-assessment score between 1 and 10 regarding their perceived level of proficiency in sarcopenia management. Responses to the above-mentioned question were also reported descriptively using frequencies (*n*) and percentages (%). The multiple-choice questions posed to the participants are designed in such a way that they can select one or more options.

### 2.3 Statistical analysis

Statistical analysis of the data was performed with IBM SPSS Statistics for Windows, version 23.0 (IBM Corporation, Armonk, NY, United States) program. The normally distributed quantitative variables are presented as mean ± standard deviation because normality was confirmed through assessments of skewness, kurtosis, their respective coefficients, and the Kolmogorov–Smirnov tests, all indicating acceptable conformity with the normal distribution. Mean ± standard deviations were used to represent the normally distributed quantitative variables. Frequency (*n*) and percentage (%) values were calculated for categorical variables. For univariate analyses, since the variables involved were categorical, chi-square tests were deemed the most appropriate method. The statistical significance level was accepted as *p* < 0.05.

## 3 Results

### 3.1 Basal characteristics

Basal demographic and clinical characteristics of patients are given in Table 1. A total of 405 family physicians, 42.0% (*n* = 170) female and 58.0% (*n* = 235) male, with a mean age of 38.05 ± 8.79 years, participated in the study. The highest level of participation was from the Southeastern Anatolia region, accounting for 32.3% of respondents. In terms of professional experience, 29.1% of participants (*n* = 118) had been working for 6–10 years, 27.4% (*n* = 111) for 16 years or longer, and 23.5% (*n* = 95) for 0–5 years, 27.7% of participants had completed a family medicine residency. The rate of participants who reported that they had not received any training about palliative care was 84%. When asked about their frequency of meeting patients aged 65 and over in the clinic, 52.8% of participants (*n* = 214) indicated ‘often,’ 36.8% (*n* = 149) ‘always,’ and 0.7% (*n* = 3) ‘rarely’ (Table 1).

**Table 1:** Distribution of participants by baseline demographics, region, and occupational characteristics.

Parameters		Mean	Std
Age		38.05	8.79
		*n*	%
Gender	Male	235	58.0
	Female	170	42.0
Region	Mediterranean	49	12.1
	Eastern Anatolia	52	12.8
	Aegean	36	8.9
	Southeastern Anatolia	131	32.3
	Central Anatolia	51	12.6
	Black Sea	30	7.4
	Marmara	56	13.8
Years in practice	0–5	95	23.5
	6–10	118	29.1
	11–15	81	20.0
	≥16	111	27.4
Family medicine speciality status	Yes	112	27.7
	No	293	72.3
Status of palliative care training	Yes	65	16.0
	No	340	84.0
Frequency of meeting patients aged 65 and over in the outpatient clinic	Occasionally	39	9.6
	Rarely	3	0.7
	Often	214	52.8
	Always	149	36.8

### 3.2 Findings on the knowledge and attitudes of family physicians about sarcopenia

With a rate of 61%, the participants reported that they had not received any training on sarcopenia, while only 3.6% (*n* = 55) indicated that they had a relative or acquaintance diagnosed with sarcopenia. Furthermore, 37.5% of respondents correctly defined sarcopenia as a “syndrome.” Conversely, 5.9% of physicians agreed with the inaccurate statement that “sarcopenia is unpreventable,” and 16.3% had no opinion on the matter. Approximately half of the participants (49.6%) appropriately disagreed with the assertion that overweight or obese individuals are at lower risk for sarcopenia compared to individuals with normal weight. The relevant results are also shown in Table 2.

**Table 2:** Distribution of participants’ responses to items measuring knowledge about sarcopenia.

Items	Responses	*n*	%
Have you got education about sarcopenia	Yes	158	39.0
	No	247	61.0
Do you have a relative or acquaintance diagnosed with sarcopenia	Yes	55	13.6
	No	350	86.4
How to identify sarcopenia	Condition	130	32.1
	Disease	82	20.2
	Syndrome*	152	37.5
	Have no idea	41	10.1
Sarcopenia is not preventable	Agree*	24	5.9
	Disagree	315	77.8
	Have no idea	66	16.3
Sarcopenia lasting less than 6 months: acute type; more than 6 months: chronic type	Agree*	275	67.9
	Disagree	9	2.2
	Have no idea	121	29.9
Overweight or obese individuals have a lower risk of sarcopenia compared to normal-weight individuals.	Agree	101	24.9
	Disagree*	201	49.6
	Have no idea	103	25.4
The presence of sarcopenia increases the risk of hospitalization and increases the cost of care during hospitalization.	Agree*	379	93.6
	Disagree	2	0.5
	Have no idea	24	5.9

*Correct answers corresponding to items measuring knowledge level about sarcopenia are highlighted in bold in the relevant column.

33.69% of the participants correctly agreed that “sarcopenia progresses with a progressive, generalized loss of muscle mass and muscle strength,” while 32.94% agreed that “it can lead to negative outcomes such as reduced muscle function, lower quality of life, and increased mortality rates.” Among the incorrect responses, 2.89% of participants selected the statement “sarcopenia is a bone disease,” and 2.25% believed that “it is only observed in older adults individuals” (data not shown in the table).

### 3.3 Findings on the diagnosis and treatment of sarcopenia

Responses to questions concerning the diagnosis and treatment of sarcopenia are presented in Table 3. For the question, “In which individuals should measurements for the preliminary diagnosis of sarcopenia be applied?,” participants responded as follows: 23.91% selected “all older adults,” 21.74% “older adults with movement problems,” 20.13% “older adults with comorbidities,” 17.55% “older adults with malnutrition,” and 16.67% “cancer patients” (All options were correct). Regarding the question, “Which criteria should be used to diagnose sarcopenia?,” 18.50% of participants chose “muscle mass measurement,” 16.87% “muscle strength measurement,” 15.47% “clinical impression,” 15.63% “physical performance assessment,” and 10.82% “frailty index.”

**Table 3:** Distribution of responses to questions regarding diagnose and treatment of sarcopenia.

Parameters	Statements	*n*	%
*In which individuals should measurements for the preliminary diagnosis of sarcopenia be applied??	Older adults with mobility problems	270	21.74
	Malnourished older adults	218	17.55
	Cancer patients	207	16.67
	Older adults with comorbidities	250	20.13
	All older adults	297	23.91
*What criterion (s) should be used to diagnose sarcopenia?	Physical performance assessment	289	15.63
	Muscle strength measurement	312	16.87
	Nutritional status	185	10.01
	Fragility index	200	10.82
	Body mass index	197	10.65
	Body mass index	342	18.50
	Clinical impression	286	15.47
	Lifestyle characteristics	1	0.05
	Laboratory data	1	0.05
	Electromyography	1	0.05
	I have no idea	35	1.89
*Which of the following definitions is used in the diagnosis of sarcopenia?	European Society of Clinical and Metabolic (ESPEN)	23	3.66
	European Study Group on Sarcopenia in the Elderly 2 (EWGSOP2)	126	20.03
	Criteria of fragility by Frieder	29	4.61
	Baumgartner 1998 Additional lean mass index	20	3.18
	Criteria for fragility by Rockwood	25	3.97
	Skeletal Muscle Mass Index	39	6.20
	Janssen 2004	39	6.20
	International Sarcopenia Working Group (IWGS)	48	7.63
	National Institutes of Health Foundation (FNIH)	16	2.54
	No idea	264	41.97
*What treatment methods and supplements should sarcopenia be treated with?	Aerobic exercises	251	13.99
	Pharmacological intervention	131	7.30
	Resistance exercises	306	17.06
	Vitamin D	286	15.94
	Protein+ Pharmacological treatment		
	Calcium	235	13.10
	Balance exercises	240	13.38
	Protein	281	15.66
	I have no idea	61	3.40

*Multiple choice questions.

Participants were asked to self-assess their competence in the management of sarcopenia on a scale ranging from 1 to 10. The mean self-rated competence score was 3.37 ± 1.98, indicating a generally low level of confidence in managing this condition (Table 4).

**Table 4:** Distribution of participants’ self-assessed competence scores in sarcopenia management.

Item	*N*	Min.	Max.	Mean	Std.
How would you rate your competence in managing sarcopenia?	405	1.00	10.00	3.37	1.98

Scores*	*n*	%
1.00	97	24.0
2.00	52	12.8
3.00	85	21.0
4.00	50	12.3
5.00	63	15.6
6.00	29	7.2
7.00	20	4.9
8.00	5	1.2
9.00	1	0.2
10.00	3	0.7
Total	405	100.0

*Self-assessed scores.

Table 5 presents the distribution of participants’ responses to questions regarding the EWGSOP2 sarcopenia diagnostic criteria. Regarding the cutoff values for low grip strength, only 9.9% correctly identified the thresholds as <27 kg for men and <16 kg for women. A substantial 84.2% indicated no opinion on this matter. Concerning calf circumference as a measure of low muscle mass, just 3.7% correctly selected <31 cm, while 86.4% had no opinion. When asked about the EWGSOP2 cutoff for slow gait speed, only 4.9% correctly identified the threshold as <0.8 m/s, with 84.7% indicating no opinion. Regarding the most reliable measure of muscle function in sarcopenia diagnosis, 27.9% correctly selected muscle strength, whereas 84.7% had no opinion.

**Table 5:** Distribution of participants’ responses to questions regarding EWGSOP2 sarcopenia diagnostic criteria.

Items	Responses	*n*	%
According to EWGSOP2, what are the cutoff values for low grip strength in men and women?	Men < 24 kg, Women < 13 kg	10	2.5
	Men < 27 kg*, Women < 16 kg*	40	9.9
	Men < 34 kg, Women < 23 kg	5	1.2
	Men < 37 kg, Women < 26 kg	9	2.2
	No idea	341	84.2
According to EWGSOP2, what is the calf circumference cutoff value for low muscle mass in men and women?	<25 cm	16	4.0
	<27 cm	17	4.2
	<29 cm	7	1.7
	<31 cm*	15	3.7
	No idea	350	86.4
According to EWGSOP2, what is the cutoff gait speed value for defining low physical performance in men and women	<0.2 m/s	16	4.0
	<0.4 m/s	15	3.7
	<0.6 m/s	11	2.7
	**<0**.8 m/s*	20	4.9
	No idea	343	84.7
In sarcopenia diagnosis, which of the following is the most reliable measure of muscle function	Physical performance	79	19.5
	Muscle strength*	113	27.9
	Muscle quality	31	7.7
	Muscle mass	69	17.0
	No idea	113	27.9

Correct answers corresponding to items measuring knowledge level about sarcopenia are highlighted in bold in the relevant column.*Expected correct answers were; Men < 27 kg, Women < 16 kg, <31 cm, <0.8 m/s, and Muscle strength (highlighted bold).

The most common challenges in the sarcopenia diagnosis, treatment, and clinical implementation were as follows: 25.30% of respondents did not perceive themselves as responsible; 28.26% reported insufficient training in sarcopenia; 15.21% lacked training specific to muscle mass measurement; and 14.85% indicated unavailability of devices to measure hand-grip strength and muscle mass. Notably, 381 family physicians (94.1%) stated that participation in this study positively enhanced their knowledge and awareness of sarcopenia. Full data are given in Table 6.

**Table 6:** Distribution of family physicians’ attitudes and reported challenges in sarcopenia management.

Items	Responses	*F*	%
“Do you utilize a standardized protocol or clinical guideline for diagnosing and managing sarcopenia in your practice?”	Yes	4	1.0
	No	401	99.0
Have you made a preliminary diagnosis of sarcopenia in your workplace in the last 1 year?	Yes	19	4.7
	No	386	95.3
What challenges do you encounter in the diagnosis, treatment, and clinical implementation of sarcopenia?*	Lack of a device to measure hand grip strength and muscle mass	165	14.85
	Lack of motivation	86	7.74
	Lack of sufficient training in sarcopenia diagnosis and management	314	28.26
	Lack of patient awareness regarding the importance of sarcopenia treatment	96	8.64
	Lack of perceived responsibility for managing sarcopenia.	281	25.30
	Lack of training in measuring muscle mass	169	15.21
Has this study contributed positively to enhancing your knowledge and awareness of sarcopenia?	Yes	381	94.1
	No	24	5.9

*Multiple choice question.

### 3.4 Chi-square analysis

Responses to questions about the definition and diagnosis of sarcopenia based on EWGSOP 2018 criteria were analyzed between the groups with chi-square analysis. Statistically significant difference was not observed between groups based on years of professional experience regarding agreement with the statement “sarcopenia cannot be prevented” (*p* = 0.177), and no significant difference was found between the parameter about absence of family medicine residency training and correct identification of EWGSOP2 cutoff values for low grip strength in men and women (*p* = 0.271). A significant difference was identified between participants’ responses to the most reliable measure of muscle function in sarcopenia diagnosis and having a relative or acquaintance diagnosed with sarcopenia (*p* = 0.012). Participants’ responses to the question “How to define sarcopenia?” differed significantly depending on whether they had received prior education on sarcopenia (*p* = 0.001). Family physicians who had received palliative care training reported significantly higher proficiency scores in sarcopenia management compared to those without palliative care training (*p* < 0.001). Significant results gained from chi-square analysis of responses to muscle function measure, education, and training variables in relation to sarcopenia questions are displayed in Table 7.

**Table 7:** Chi-square analysis of responses to muscle function measure, education, and training variables in relation to sarcopenia questions.

Items		*Which of the following is the most reliable measure of muscle function in the diagnosis of sarcopenia?*						Chi-square test	
		PP *n* (%)	MS *n* (%)	MQ *n* (%)	MM *n* (%)	No idea *n* (%)	Total *n* (%)	*χ* ^2^	*p*
Presence of a relative or acquaintance diagnosed with sarcopenia	Yes	11 (20.0%)	16 (29.1%)	6 (10.9%)	16 (29.1%)	6 (10.9%)	55 (100.0%)	12.935	0.012
	No	68 (19.4%)	97 (27.7%)	25 (7.1%)	53 (15.1%)	107 (30.6%)	350 (100.0%)		
	Total	79 (19.5%)	113 (27.9%)	31 (7.7%)	69 (17.0%)	113 (27.9%)	405 (100.0%)		

Items		*“How to identify sarcopenia?”*					Chi-square test	
		Condition *n* (%)	Disease *n* (%)	Syndrome *n* (%)	No idea *n* (%)	Total *n* (%)	*χ* ^2^	*p*
Having education about sarcopenia	Yes	44 (27.8%)	37 (23.4%)	71 (44.9%)	6 (3.8%)	158 (100.0%)	16.772	0.001
	No	86 (34.8%)	45 (18.2%)	81 (32.8%)	35 (14.2%)	247 (100.0%)		
	Total	130 (32.1%)	82 (20.2%)	152 (37.5%)	41 (10.1%)	405 (100.0%)		

Items	*“How would you rate your level of competence in managing sarcopenia?”*				Independent-sample *t*-test	
		*N* (%)	Mean	Standard deviation	*t*	*p*
Previous palliative care training	Yes	65 (16.0%)	4.29	2.01	4.202	0.000
	No	340 (84.0%)	3.19	1.92		

PP, physical performance; MS, muscle strength; MQ, muscle quality; MM, muscle mass.

## 4 Discussion

Our research on sarcopenia awareness, knowledge, and clinical practices of family physicians in Türkiye demonstrated notably low familiarity with the concept of sarcopenia with only a small fraction reporting formal training in this subject. Supporting our assumption, the presence of individuals diagnosed with sarcopenia in the immediate environment and the receipt of relevant training revealed a significant difference in knowledge and awareness between the groups. In the presented study, most participants had not previously received training on sarcopenia or palliative care.

In addition, the ability to recognize diagnostic criteria and use sarcopenia-specific tools was significantly below the expected levels. Limited exposure to specialized diagnostic tools (such as grip strength tests or gait assessments) in general practice may reduce familiarity. Furthermore, without standard definitions or attainable screening tools, sarcopenia remains less visible in everyday clinical workflows (23, 24, 33). The complexity and resource requirements of many diagnostic tools (e.g., DEXA and BIA) hinder their adoption in general practice. While measuring handgrip strength is inexpensive and practical, equipment such as dynamometers is not widely available in primary care settings, and alternatives such as chair stand or gait speed tests, despite their simplicity, are underutilized (24, 27, 28).

The findings of the presented study point to a fundamental issue that sarcopenia is underrecognized or overlooked in primary care. Like many healthcare professionals from different regions (34, 35). The findings of the presented study also revealed the need for training of family physicians on sarcopenia. Promisingly, the widespread rejection of the phrase that sarcopenia is inevitable among family physicians in the current study points to a strong basic understanding and, importantly, a professional readiness to take responsibility for sarcopenia prevention and proactively incorporate preventive strategies into routine primary care. Regional and professional differences in familiarity with sarcopenia diagnostic tools often stem from variation in training opportunities, the clinical prominence of geriatric medicine, and standardized practice environments. For instance, specialists such as geriatricians and rehabilitation physicians are more likely to learn and apply sarcopenia concepts regularly, whereas primary care providers and family physicians often have less exposure (19). Compared to earlier studies by Reijnierse (34) and Yeung (35), knowledge on this issue appears to be more widespread among our respondents, suggesting some progress in awareness. However, the proportion of physicians who reported having preliminarily diagnosed sarcopenia in the past year remains remarkably low highlighting a persistent gap between theoretical understanding and clinical application.

Initially, training interventions yield substantial gains in healthcare professionals’ belief in the preventability of sarcopenia and their familiarity with its diagnostic criteria. However, it cannot be asserted that the impact of one-time training interventions that are not supported by feedback and continuous training will be sufficient. This pattern aligns with Reijnierse et al.’s (34) work, where sarcopenia training significantly enhanced conceptual understanding and diagnostic tool usage specifically walking speed, grip strength, and muscle mass measurement. Yet, although this reflects positive learning outcomes, it is concerning that these newly acquired competencies appear to decrease unless actively reinforced. Yeung et al.’s (35) multicenter study similarly demonstrated dramatic immediate improvements in both sarcopenia awareness and the correct application of gender-specific grip strength thresholds immediately after training. However, 6 months later, these competencies had diminished markedly, highlighting a significant disparity between short-term learning and long-term retention” (35). Guralnik et al.’s (19) survey underscores a significant gap in the recognition and application of sarcopenia-related terminology among internists and family medicine physicians. A substantial proportion of these practitioners report limited familiarity with the term “sarcopenia,” and many do not consistently employ standardized diagnostic criteria. Instead, they often default to nonspecific terms such as “muscle loss” or “deconditioning,” which lack precise clinical definitions. This trend highlights a broader issue of conceptual ambiguity and a lack of standardization in the diagnosis and management of sarcopenia within primary care settings. The absence of a unified definition and diagnostic framework for sarcopenia may contribute to its underdiagnosis and undertreatment, emphasizing the need for enhanced education and consensus among healthcare providers.

In the current study, most family physicians were identified as mid-career professionals, and only a minority had completed formal residency in family medicine. Ter Beek et al.’s (36) multicenter study similarly highlighted that dietitians are often early in their professional journeys, and few are active in primary care. Both data sets converge on a critical finding: comprehensive understanding of sarcopenia remains limited. Interestingly, those working in institutional settings, such as hospitals or long-term care facilities, had a relatively stronger knowledge base. Importantly, however, neither years of experience nor professional tenure were reliable predictors of sarcopenia awareness (36). These findings suggest that clinical exposure alone does not guarantee preparedness in recognizing or managing sarcopenia. Therefore, effective educational strategies must reach across the spectrum of experience and workplace settings, ensuring that regardless of background or career stage, healthcare professionals gain the depth of understanding required to address sarcopenia proactively in their practice.

In current study it reveals that witnessing sarcopenia cases in the immediate vicinity strengthens the level of awareness and knowledge on the subject. As a matter of fact, in our study, the presence of individuals diagnosed with sarcopenia in the immediate environment revealed a positive significant difference in terms of sarcopenia management competence scores. A study by Kiss et al. (37) on healthcare workers in cancer clinics showed that there is a high level of awareness and knowledge about cancer-associated sarcopenia. In their study, majority of the clinicians were able to accurately evaluate the definition of sarcopenia and its importance in cancer treatment (37). These results can be interpreted to mean that malnutrition is common in cancer patients and oncologists are familiar to sarcopenia.

Our study suggests that while awareness of sarcopenia in obese individuals is improving among family physicians, actual diagnostic activity remains limited. Greater clarity in definitions, improved education, and better screening mechanisms could help bridge this gap—ultimately promoting more timely identification and management of sarcopenic obesity. The findings of Nimphan et al. (38) underscore the importance of recognizing sarcopenia even in the context of obesity among chronic stroke patients. This challenges the common assumption that higher body weight confers protection against muscle degeneration. Correspondingly, our survey of family physicians revealed that approximately half did not believe overweight or obese individuals are less prone to sarcopenia compared to their normal-weight counterparts. Moreover, there was near-unanimous agreement that sarcopenia contributes to higher hospitalization rates and elevates healthcare costs (38).

A major challenge complicating both diagnosis and awareness is the lack of a universally accepted definition of sarcopenia. This variability likely contributes to inconsistencies in identification and reporting across different settings. Addressing this issue requires not only broader consensus on diagnostic criteria but also enhanced training and practical tools to support clinicians in recognizing sarcopenia—particularly in patients who also present with obesity. The absence of universally adopted diagnostic criteria—despite efforts by groups like EWGSOP, Asian Working Group For Sarcopenia (AWGS), and Foundation for the National Institutes of Health (FNIH)—leads to fragmentation in both teaching and clinical practice. Different settings may favor one tool over another, leading to inconsistent awareness and use (39).

When the literature is reviewed, it is seen that although there are some studies conducted in clinics in Türkiye, especially on the older population who are in the sarcopenia risk group, there are also a small number of community-based prevalence studies and the prevalence of sarcopenia may differ according to the diagnostic criteria used and studies have shown that not focusing on this issue, not knowing diagnostic criteria and not using tools for diagnosis are barriers to ideally management of sarcopenia (40–42). In several studies small part of participants correctly answered the diagnostic criteria related items compared to current study (19, 35, 36). At this point, there is also a need for a national definition standard to accurately determine the prevalence of sarcopenia in Türkiye. Since the definition of sarcopenia cannot be standardized at the international level, it is inevitable that there will be difficulties in diagnosis and differences between diagnosis (43).

Sarcopenia can be attenuated or even reversed through a thoughtful combination of physical training and diet optimization. Implementing such integrative strategies in clinical practice carries significant potential to preserve mobility, autonomy, and quality of life in aging populations (7, 12, 15). The effectiveness of dietitian participants in nutrition monitoring and energy planning and the fact that physiotherapists are more effective in physical performance and function evaluation points reveal the need for a multidisciplinary approach in the management of sarcopenia. The adoption of this approach by primary care physicians can be considered an important factor for success in the management of sarcopenia (44). Our study reveals a limited level of awareness regarding key interventions for sarcopenia, such as resistance training, vitamin D supplementation, and protein support, among family physicians. This mirrors previous research indicating that familiarity with sarcopenia remains limited among internists and primary care practitioners, whereas geriatricians and rehabilitation specialists tend to display greater recognition of the condition (19). Interestingly, the few physicians in our sample who had undergone palliative care training often demonstrated stronger competency in sarcopenia management. This aligns with the broader notion that targeted clinical education especially when it relates to older adults’ care can significantly enhance practitioners’ readiness to address sarcopenia. Notably, earlier surveys highlight that a small minority of internal medicine and family physicians report familiarity with the term “sarcopenia,” while geriatricians and physical medicine specialists report considerably higher awareness (19, 34). This underscores a systemic gap: although the condition is prevalent in older adult populations seen in primary and internal care, it is not yet fully embraced within routine professional practice. Compounding this issue, even among geriatrics clinicians, there is evidence that proficiency in related geriatric assessments such as identifying malnutrition risk or fall vulnerability often falls short of expectations (45, 46) Similarly, multidisciplinary reviews from various countries highlight widespread gaps in knowledge about sarcopenia across health professions, except for oncology specialists who appear comparatively better informed (22).

Collectively, our findings and those from the literature show that positive attitudes toward sarcopenia may exist, but they are not consistently matched by actionable knowledge or practice. Primary healthcare inherently relies on collaborative teamwork, and nurses play a critical role in the early recognition and management of sarcopenia. A study from Thailand reported that over half of the participating nurses correctly identified the term “sarcopenia,” yet their practical awareness around its management remained limited (47). This finding underscores that healthcare outcomes improve when structured, cross-disciplinary frameworks support knowledge translation into action. Education and collaborative clinical environments appear essential particularly in primary care settings where nurses often serve as frontline responders to geriatric syndromes.

Undoubtedly, for success in the fight against sarcopenia, there is a need for healthcare providers to increase the knowledge and awareness of especially risky patient groups through effective communication and patient education.

## 5 Strength and limitations

This study has some limitations. Although the number of respondents met and slightly exceeded the statistical requirement for evidential value, we acknowledge that a larger sample size would have further strengthened the representativeness of the findings. Several elements may have influenced participation in the study. The voluntary nature of involvement and the absence of any obligation to respond may have contributed to a lower response rate. In addition, limited awareness regarding the importance of the research topic and intrinsically lower motivation within the field may have played a role. Moreover, the demanding workload and time constraints typically faced by primary care physicians may have further affected participation levels. Furthermore, reliance on an online survey platform may have posed accessibility challenges such as infrastructure limitations and unstable internet connectivity that could have hindered participation from certain individuals. Since there is no scale developed or validated in Türkiye on this subject, the data collection form was created by the researchers and no scores could be assigned to the answers of the participants. Taken together, these factors can be considered to have contributed to the underrepresentation of certain regions in the study. Nonetheless, this study represents the first research conducted among primary care family physicians in Türkiye on this topic. Nearly all physicians participating from different regions confirmed an increased awareness of sarcopenia through the survey questions. Furthermore, the study enabled the identification of educational needs and the challenges in the field. Therefore, we believe these findings can contribute to raising awareness and help guide future improvement plans and strategic initiatives in this field.

In conclusion, the findings of this study revealed the need for raising awareness about sarcopenia in primary care in Türkiye by pre- and post-graduate trainings. Family physicians should be supported by guidelines suitable for primary care and by equipment support for screening and diagnosis. Achieving a global consensus on the threshold values used in the diagnosis of sarcopenia and adapting them to societies ideally, infrastructure studies for the establishment of multidisciplinary support teams in the management of sarcopenia, the use of informative public spots on all media tools and social platforms to increase the awareness of the society, and the motivated efforts of physicians in this regard can provide significant gains in the fight against sarcopenia. We believe that studies focusing on sarcopenia will make a positive contribution in the context of preventing deterioration in quality of life due to falls and fractures, especially in the older population, and reducing health costs due to hospitalizations.

## Data Availability

The raw data supporting the conclusions of this article will be made available by the authors, without undue reservation.
